# Supporting sustainable long-term residential care in Ireland: a study protocol for the Sustainable Residential Care (SRC) project

**DOI:** 10.12688/hrbopenres.13543.1

**Published:** 2022-04-12

**Authors:** Brendan Walsh, Sheelah Connolly, Maev-Ann Wren, Leonie Hill

**Affiliations:** 1 Economic and Social Research Institute, Dublin, Ireland; 2 Trinity College Dublin, Dublin, Ireland

**Keywords:** Long-term residential care (LTRC), COVID-19, Ireland, Governance, Ownership.

## Abstract

The coronavirus disease 2019 (COVID-19) pandemic brought to the fore deficiencies in the long-term residential care (LTRC) sector, including issues of governance, funding and staffing. Many of these issues pre-dated the pandemic and have contributed to concerns around the sustainability of the current model of LTRC in Ireland. The aim of the project detailed in this protocol is to provide an evidence base to help ensure the sustainability of the LTRC sector in Ireland within a new wider model of care for older people. The project includes three key objectives: (i) to describe and analyse the characteristics of LTRC homes across Ireland; (ii) to examine the association between LTRC home characteristics and COVID-19 outbreaks and deaths and (iii) to identify challenges to the sustainability of the LTRC sector within a COVID-19 environment and beyond. Bringing together the findings from these three objectives, the project will identify approaches and strategies which will help ensure the sustainability of LTRC that meets the needs of residents. The proposed research incorporates quantitative analyses and a review. Combining data from a variety of administration sources and using a variety of statistical techniques, the project will include a retrospective observational analysis of COVID-19 in LTRC homes in Ireland. Subsequently, a review will examine the current funding model of LTRC in Ireland, as well as the regulations and governance structure that underlie the system. The review will also examine international practices in these areas. Bringing together the findings from the quantitative analysis and the review and working with the knowledge users on the project, the project will build upon recent work in the area to identify the current challenges to the system of LTRC and possible solutions.

## Introduction

### Long-term residential care (LTRC) in Ireland

While the definition and systems of long-term residential care (LTRC) differ across countries, it generally refers to long-term care given to people who reside in a residential setting. In Ireland, LTRC forms a central component of the care of older people. In 2019, an estimated 32,000 people lived in LTRC settings, of whom 94% were aged 65 years and older ( Walsh *et al*., 2021). The total expenditure on LTRC in Ireland in 2019 was almost €2 billion, making it one of the largest components of total health and social care spending ( Walsh *et al*., 2021).

Existing LTRC homes differ along several domains including the form of ownership with three main categories – public (Health Service Executive (HSE)), voluntary (private ‘not-for-profit’) and private (‘for-profit’). Public and voluntary institutions were the predominant providers of residential care up to the early 2000s. Since then, the private sector has become the dominant provider, in part driven by tax incentives introduced to encourage an increase in private provision, and in part by the establishment of the Nursing Homes Support Scheme (NHSS, ‘Fair Deal’) which provided increased financial security for the sector. Meanwhile, public providers were affected by fiscal constraints, public employment ceilings and regulatory requirements for upgraded facilities ( Mercille, 2018; Wren *et al*., 2017). In 2020, beds in privately owned care homes constituted 78% of all LTRC beds ( HIQA, 2020a). In recent years, a number of international private operators have entered the Irish LTRC market with foreign capital having acquired part, or all, of eight of the largest 15 private operators in the country in recent years ( Kehoe, 2021). Under the Health Act (2007), all LTRC homes must register with the Health Information and Quality Authority (HIQA) and comply with the conditions and requirements set by HIQA ( Wren *et al*., 2017). HIQA can inspect homes for registration purposes and to ensure quality standards. 

The majority of LTRC in Ireland is privately provided but publicly funded ( Walsh *et al*., 2021). The NHSS, a statutory scheme for LTRC that confers access to residential support for those who require it, was established in 2009. At the end of 2019, approximately 73% of LTRC residents were funded under the NHSS. Those funded through the NHSS are required to make a co-payment for care based on an assessment of income and assets (including the family home) ( Wren *et al*., 2017); the co-payment is up to 80% of assessable income and up to 7.5% (per annum) of the value of any assets above €36,000. Those in receipt of the NHSS can access care in any public, voluntary or private care home that is participating in the NHSS scheme. The average cost of providing an NHSS-funded bed per annum is €61,021, with the State contributing 69% of the costs ( Walsh *et al*., 2021).

### COVID-19 and the LTRC sector

The LTRC sector has been significantly impacted by the coronavirus disease 2019 (COVID-19) pandemic. A significant proportion of LTRC settings internationally have reported COVID-19 outbreaks, with high rates of morbidity and mortality and high rates of staff absenteeism ( European Centre for Disease Prevention and Control, 2020). In many countries, COVID-19 deaths in LTRC residents accounted for over half of all COVID-19 deaths ( Comas-Herrera *et al*., 2021). Countries such as Australia (75%), Canada (59%), Belgium (57%) and the Netherlands (51%) had particularly high proportions of COVID-19 deaths occurring amongst LTRC home residents ( Comas-Herrera *et al*., 2021). The percentage of people diagnosed with COVID-19 who died from the illness (case fatality rate) in LTRC settings was also found to be much higher than in the community, with case fatality rates of 36% in the UK in 2020 ( Dutey-Magni *et al*., 2021). Research from Canada found that the COVID-19 fatality rate in LTRC residents was 13.1 higher than the rate for community-living adults older than 69 years ( Fisman *et al*., 2020). Ireland also saw very high rates of COVID-19 infections and mortality amongst LTRC home residents. From March 2020 to end of November 2021, 62% of COVID-19 deaths were linked to outbreaks in nursing homes, with 4.1% linked to other community hospital and long stay settings. Due to the older age and high level of morbidity among residents in LTRC settings, the impact of a COVID-19 outbreak can be significant; in addition, the movement of LTRC staff across homes can contribute to the spread of the virus ( McMichael *et al*., 2020).

### Factors associated with outbreaks in the LTRC sector

Since the beginning of the pandemic, a number of studies have been undertaken looking at the factors associated with COVID-19 outbreaks in the LTRC sector, with wide-ranging and somewhat conflicting results. In Ireland, limited analyses have taken place on the subject. An analysis of COVID-19 outbreaks in LTRC homes between March and November 2020 found that the probability of an outbreak was positively associated with increasing incidence in the community close to the home and to the number of beds in the home ( HIQA & HPSC, 2021). No relationship was found between ownership type (e.g. public, private) and the probability of an COVID-19 outbreak ( HIQA & HPSC, 2021). Relative to the first wave of COVID-19 in Ireland (1 March to 1 August 2020), there were fewer outbreaks in LTRC settings in the second wave (2 August to 21 November 2020) and when they did occur, they tended to be smaller in terms of the number of people infected ( HIQA & HPSC, 2021).

The factors associated with COVID-19 outbreaks in LTRC differ across countries. In England, for example, Shallcross *et al*. (2021) found an increased probability of outbreaks in LTRC homes that were for-profit (relative to those that were not-for-profit), in homes where there was frequent employment of agency nurses or carers, and in homes that reported difficulties in isolating patients. A US study found that larger staff size was linked to a higher number of COVID-19 cases in 2020; however, staffing quality measures (including direct care staff-to-resident ratios and skill mix), were not significant predictors of COVID-19 cases or deaths ( McGarry *et al*., 2021). Abrams *et al*. (2020) examined the characteristics of LTRC homes in the US with COVID-19 cases in the early months of the pandemic and found that larger size, urban location, greater percentage of African American residents, non-chain status, and state were significantly related to the increased probability of having a COVID-19 case. Ownership type was not found to be significantly related ( Abrams *et al*., 2020). A study from Italy examined the relationship between geography, size, design, organizational characteristics, and implementation of infection prevention and control measures and the extent of COVID-19 outbreaks in LTRC homes between March and May 2020 ( Cazzoletti *et al*., 2021). They found that incidence of outbreaks was not significantly associated with any of the variables, except for geographical region.

To date a small number of review studies have been undertaken seeking to collate existing studies which have examined the factors associated with COVID-19 outbreaks in the LTRC sector. For example, a literature review including 18 studies examining the relationship between the ownership status of LTRC homes and their outcomes during the early months of the pandemic noted that the majority of studies found an association between ownership status and effectiveness in response to the pandemic ( Kruse *et al*., 2021). However, this association was attenuated when other factors (including size, staff shortages and regional spread of COVID-19) were included in the analysis. A later systematic literature review examined the associations between LTRC home ownership and COVID-19 outbreaks, infections and mortality and included 32 studies across five countries ( Bach-Mortensen *et al*., 2021). The review found that private (for-profit) homes did not consistently have an elevated risk of COVID-19 outbreaks; however, such homes did tend to have higher rates of infections and deaths. Seeking to explain this finding, the review found that private (for-profit) ownership was associated with shortages of personal protective equipment (PPE) ( Bach-Mortensen *et al*., 2021). The review concluded that the variation in COVID-19 outcomes was not explained by ownership type alone and that other factors related to staffing, size and resident characteristics are also associated with poorer outcomes. The research outlined in this protocol will build upon this international literature to identify those factors most associated with COVID-19 outbreaks and deaths in LTRC in Ireland.

### State response to COVID-19 in the LTRC sector

Many observers have argued that there was insufficient priority placed on safeguarding the LTRC sector at the beginning of the pandemic in Ireland ( Pierce *et al*., 2020) and internationally ( Comas-Herrera *et al*., 2020; Grabowski, 2020). An Expert Panel on Nursing Homes was convened in 2020 to examine the LTRC sector’s response to the pandemic. In a report, the Expert Panel highlighted that “ *at the beginning of the pandemic, efforts were made to ensure that sufficient acute hospital capacity was available, which included discharging patients who were medically fit where possible, including discharges of patients to nursing homes*” ( Frazer *et al*., 2020). This prioritisation of acute hospitals was also seen in terms of discharging patients, some of whom had COVID-19 symptoms, into LTRC, and the initial rationing of PPE in the LTRC setting.

The COVID-19 pandemic raised a number of challenges for the LTRC sector in Ireland due to the frailty of LTRC residents, the close contact needed between carers and residents, and the physical layout of many LTRC homes. Consequently it was necessary for the State to take a more proactive role in LTRC settings which included providing PPE, testing, clinical advice, staff and preparation and implementation of COVID-19 action plans ( Department of Health, 2020). Privately owned LTRC settings have no formal clinical governance links with the HSE ( HIQA, 2020b) and consequently the additional measures required to contain the spread of COVID-19 were difficult to implement in these settings in the early stages of the pandemic ( Pierce *et al*., 2020). It became necessary for the HSE to provide staffing and infection control support for the LTRC sector in response to the pandemic, prompted by an emerging wave of infection. From April 2020 to December 2021, the HSE administered the Temporary Assistance Payment Scheme (TAPS), providing private and voluntary care homes up to €60,000 per month (initially €75,000) for cleaning/infection prevention and control, social distancing and isolation facilities, and recruiting and training staff. TAPS became one of the key elements of the State’s response to the pandemic in LTRC and was the largest injection of funds specifically targeting private LTRC homes to date. In this analysis. we will examine the extent to which TAPS was used, prevented future COVID-19 outbreaks, and could be leveraged in future funding mechanisms within the sector.

### Rationale for the study

Both Irish and international research has repeatedly found that most older people prefer to remain in their own home as they age ( Abramsson & Andersson, 2016; Fox *et al*., 2017). However, even with significant home care supports, there are some older people who will require residential care when their care needs can no longer be met in the home or community ( HIQA, 2016). Despite measures aimed to facilitate people to remain within their own home, the demand for such care in Ireland is likely to increase significantly in the coming years due to the projected increase in the number of older people ( Walsh *et al*., 2021).

The COVID-19 pandemic brought to the fore deficiencies in the LTRC sector in Ireland and beyond. The Expert Panel on Nursing Homes identified a number of concerns about the sector ( Frazer *et al*., 2020) including a lack of an overarching governance structure within the sector, both within public and private homes. In addition, the Expert Panel’s report noted that there were issues with the current funding model which failed to recognise and compensate for the complex needs of some people within the LTRC setting ( Frazer *et al*., 2020); currently NHSS funding is not linked with frailty, morbidity, or cognitive impairment rates of a home’s residents. Also highlighted was the need to define staff ratios and skill mix and reduce the disconnect between regulation and oversight in the private LTRC sector in Ireland. A number of recommendations to ensure the provision of safe, quality care for Ireland’s ageing population were made by the Expert Panel ( Frazer *et al*., 2020). The research detailed in this protocol seeks to build on the analysis and recommendations from the Expert Panel to ensure a sustainable LTRC sector in Ireland.

## Aim of this research

The aim of the Sustainable Residential Care (SRC) project is to provide an evidence base to ensure the sustainability of the LTRC sector in Ireland and beyond. Throughout the project, researchers will engage with knowledge users and stakeholders to ensure that the project delivers policy-orientated outputs. In this project sustainability will be considered from a number of perspectives including the resident and their family, the LTRC workforce, LTRC providers and the State. The research seeks to identify what funding models, regulations, governance structure, etc can be used to ensure high quality and safe care for LTRC residents.

The impact of the COVID-19 pandemic on the LTRC sector in Ireland will be used as a case study. The research will examine the sector within the context of COVID-19 to better understand the current challenges to the sector and to identify what changes may be necessary to ensure a sustainable LTRC sector. The research will examine a key State intervention into the LTRC sector, TAPS, to investigate whether the scheme reduced the impact of COVID-19, and to what extent the scheme could be used as a template for future funding of the sector. Across the key deliverables of the research, the project will also seek to frame LTRC within a wider model of care of older people.

## Objectives of this research

1.   To describe and analyse the characteristics of LTRC homes across Ireland.

2.   To examine the association between LTRC home characteristics and COVID-19 outbreaks and deaths across Waves 1-3 of the COVID-19 pandemic.

3.   To identify challenges to the sustainability of the LTRC sector in Ireland within a COVID-19 environment and beyond. This will include identifying governance, provision and funding approaches that will ensure the sustainability of LTRC in Ireland using best practice from Ireland and international systems. A case study of the largest COVID-19 intervention in the sector, the TAPS, will be undertaken to garner how such schemes could be used to inform future policies.

## Study design

This research incorporates a retrospective observational analysis of COVID-19 in LTRC homes in Ireland, and a review of funding, regulation, and governance structures in LTRC in Ireland and internationally.

## Population, concept, and context

This research seeks to examine COVID-19 and sustainability in the LTRC sector in Ireland. This study examines COVID-19 in residential care settings for older people. We define the set of LTRC homes to be included in this study as the 572 homes registered with HIQA as providing residential care for older people in July 2021.

## Methods

This study involves three specific elements or objectives. We outline the specific methods for each objective below.

### Objective 1 - Overview of LTRC in Ireland

Objective 1 will describe and analyse the characteristics of LTRC homes across Ireland and discuss how the sector has evolved in recent years.

- **Data source**: Data on LTRC home characteristics will be obtained from the HIQA Bed Registry, the National Treatment Purchase Fund (NTPF), and the HSE. Population data on the number of people aged 65 years and older in each county (obtained from the CSO) will be used to compare relative supply across counties.
- **Data analyses**: Comparative descriptive statistics will be used to describe differences in LTRC homes by size, provider-type, and other relevant characteristics identified within the data. The work will first present the characteristics of LTRC homes in Ireland according to provider type, ownership structure, and size. The analyses will present regional variation in LTRC supply (per local population aged 65 years and older) and provider type across counties in Ireland. This objective will build upon previous analysis of LTRC supply across regions in Ireland by members of the research team – see Smith *et al*. (2019). Analysis will be undertaken using Stata 17 (RRID:SCR_012763).

### Objective 2 - Examination of COVID-19 in LTRC

Objective 2 will examine the association between LTRC home characteristics and COVID-19 outbreaks and deaths. In doing so the analysis will identify if particular types of homes were more or less likely to experience a COVID-19 outbreak and deaths.

- **Data source**: Data on COVID-19 outbreaks and deaths in LTRC care between 28th February 2020 and 31st March 2021 will be obtained from the HPSC’s Computerised Infectious Disease Reporting (CIDR) database that was used to capture information on COVID-19 in Ireland. The CIDR outbreak identification data includes information on date of the first and last notified COVID-19 confirmed case, number of cases in the LTRC home (partitioned by number of cases amongst residents and healthcare workers), and length of the outbreak. Length of outbreak will be measured from the date of the earliest confirmed case and 28 days after the last reported date. This follows previous analyses using similar data ( HIQA & HPSC, 2021). We will also obtain information on LTRC home characteristics from the HIQA bed register. This information will be merged with the CIDR data using the LTRC home centre ID captured in both the HIQA and CIDR data. Including the data from HIQA will allow us to include information on LTRC homes with no COVID-19 outbreaks who are not included in CIDR. The HIQA data provides information on the LTRC home provider which allows the categorisation of homes as public (HSE) or private (includes both for-profit and not-for-profit providers). Information on the maximum occupancy will be used as a proxy for the size of the home. The location information of each LTRC home will allow us to model the impact of COVID-19 rates in local areas and in neighbouring LTRC homes. Finally using publicly available data from the NTPF, we will include information on the average weekly funding for an NHSS bed for each LTRC home.
  A key limitation of the data is the lack of information on facilities and patients within LTRC. For example, no information on morbidity, dementia or cognitive status of patients is available. This makes it difficult to allocate resources based upon need through the NHSS. It also makes analysis such as ours less complete as resident-level characteristics which are likely to be to correlated with COVID-19 outcomes are unavailable ( House & Fewster, 2020).
- **Data analysis:** A two-step (hurdle) multivariate fixed effects regression model will be used to examine the association between LTRC characteristics and COVID-19 outcomes. A number of control variables will be included, including LTRC size, provider-type (e.g. HSE, voluntary, for-profit), and Nursing Home Support Scheme (NHSS) funding. To account for variations in COVID-19 outbreaks across geographic areas and time, in both steps, LTRC home fixed effects and time-varying region effects will be included. The date of the first confirmed case in neighbouring LTRC homes/healthcare facilities will be included in lieu of the first confirmed cases (if any) within respective LTRC homes.
  In step one, a probit regression will be used to compare LTRC home characteristics across our two binary outcome measures: any COVID-19 outbreaks; any COVID-19 deaths.
  In step two, a linear or negative binomial regression (based upon model fit) will be used to analyse the association between LTRC home characteristics and a count (or rate) of COVID-19 infection and deaths. Standard errors will be clustered at the level of LTRC homes. Where appropriate, analyses will be undertaken across each distinctive wave separately. Analysis will be undertaken using Stata 17 (RRID:SCR_012763).

### Objective 3 - Sustainability of LTRC

This objective seeks to identify current challenges to the LTRC sector and provide recommendations on policies to inform sustainability in the sector.

First, a review of the funding models, regulations and governance structure that currently exist in Ireland will be undertaken. Working with all knowledge users on the project, this will draw upon recent analyses of the sector including the Expert Panel on Nursing Homes Report ( Frazer *et al*., 2020) and the Department of Health’s ‘A value for money review of nursing home care costs’ report ( Department of Health, 2021). We will also identify what funding models, regulations, and governance structure are identified as best practice in other jurisdictions that could be used to ensure high quality and safe care for LTRC residents in Ireland.

Second, we will examine the largest investment into LTRC homes undertaken during COVID-19, the Temporary Assistance Payment Scheme (TAPS). This scheme was introduced in April 2020 to aid private LTRC homes. Up to €75,000 per month was initially provided for cleaning/infection prevention and control, social distancing and isolation facilities, and recruiting and training staff. We examine the extent to which TAPS was used and whether TAPS was associated with lower rates of COVID-19 outbreaks and deaths.

- **Data source**: Data will be obtained from the Department of Health on LTRC homes that availed of the TAPS. Information will be provided on whether funding from the scheme was received, the amount provided, length of assistance (months), and how costs were used (e.g. agency staff, cleaning/infection prevention and control). As TAPS was provided on a monthly basis, a longitudinal dataset at the LTRC home–month level will be constructed. Data on LTRC characteristics and COVID-19 outcomes collated for Objectives 1 and 2 will be matched to this dataset based upon LTRC centre ID which is available across datasets.
- **Data analyses**: First, a descriptive analysis on the use of TAPS across LTRC homes will be undertaken. Second, multivariate probit regression statistics will be used to examine the characteristics of LTRC homes that received any TAPS funding. Third, for those LTRC homes that received TAPS funding, separate multivariate generalised linear model regression statistics will be used to examine the association between LTRC characteristics and the amount of TAPS funding that was provided, and how funding was used. In accordance with data analyses in Objective 2, LTRC fixed effects, time varying region effects, and the date of the first confirmed case in neighbouring LTRC home/healthcare facility will be included in the regressions. Analysis will be undertaken using Stata 17 (RRID:SCR_012763).

## Study status

**Data has been obtained from the relevant data custodians and is currently being analysed.**

## Dissemination

The study findings will be disseminated through peer-reviewed open access ESRI reports and open access international journal articles. The findings will also be disseminated through the various ESRI platforms including social media updates as well as press releases and updates on the ESRI website. The study team will present findings at national and international meetings and conferences. In addition, study findings will be shared with relevant stakeholders at the earliest opportunity.

## Discussion

The LTRC sector in Ireland and internationally has been significantly impacted by the COVID-19 pandemic. In Ireland and other countries, COVID-19 deaths amongst LTRC residents made up the majority, or a large percentage of all COVID-19 deaths ( Comas-Herrera *et al*., 2021). In addition, the COVID-19 pandemic has brought to the fore issues within the LTRC sector ( Frazer *et al*.. 2020; Pierce *et al*., 2020). Consequently, the Expert Panel on Nursing Homes convened to examine the issues surrounding the management of COVID-19 among nursing homes made a number of COVID-19 specific and more general recommendations in relation to the LTRC sector ( Frazer *et al*., 2020). These included greater integration of private nursing homes with the wider framework of public health and social care and the development and roll out of standardised care needs assessment. Building on the work of the Expert Panel, the SRC project will provide an evidence base to reform the LTRC sector in Ireland to ensure a sustainable system providing high quality and safe care to those who need it.

This research will examine the current LTRC sector and investigate the association between LTRC home characteristics and COVID-19 outbreaks and deaths across Waves 1-3 of the pandemic. The research will focus on whether the largest State intervention into LTRC during the pandemic, TAPS, reduced the impact of COVID-19 in LTRC and whether certain attributes of the scheme may inform future governance of the sector. These findings will provide a context of analyses into approaches that ensure the sustainability of a LTRC sector within a wider model of care for older people. In this sense, the research will work with knowledge users to identify governance, provision and funding approaches that will ensure the sustainability of LTRC in Ireland using best practice from Ireland and international systems.

## Data availability

No data are associated with this article.
