# Supplementary material for: Supporting sustainable long-term residential care in Ireland: a study protocol for the Sustainable Residential Care (SRC) project
Source: HRB Open Res. 2022 Apr 12;5:30. [Version 1] doi: 10.12688/hrbopenres.13543.1 (PMC9086518; doi:10.12688/hrbopenres.13543.1)
Supplement: Peer review history

# Peer review: Reviewer response for version 1

**DOI:** 10.21956/hrbopenres.14783.r31866

**Published:** 2022-05-09

**Authors:** Maria Pierce

**Affiliations:** 1 School of Social Policy, Social Work and Social Justice, University College Dublin, Dublin, Ireland

This article is a study protocol outlining the design of a study, the Sustainable Residential Care project, aiming to provide an evidence base to help ensure the sustainability of the long-term residential care (LTRC) sector for older people in Ireland.

 The introductory section of the article succinctly but clearly describes the LTRC system for older people in Ireland, providing an overview of ownership, regulation and financing. 

 The section on COVID-19 and the LTRC sector summarises international and Irish evidence showing that LTRC settings for older people were disproportionately impacted by the COVID-19 pandemic. The discussion on factors associated with outbreaks of COVID-19 in LTRC settings shows that factors are wide-ranging, differ across countries, and that results are somewhat conflicting and thereby highlights the importance of understanding which factors were most associated with COVID-19 outbreaks and deaths in Ireland. This is followed by a summary of the State’s response to COVID-19 in the LTRC sector.

 The study has three main objectives: 

- Objective 1: To describe and analyse the characteristics of LTRC in Ireland.
- Objective 2: To examine the association between LTRC home characteristics and COVID-19 outbreaks and deaths across Waves 1-3 in Ireland.
- Objective 3: To identify challenges to the sustainability of the LTRC sector in Ireland within a COVID-19 environment and beyond. This will include identifying governance, provision and funding approaches that will ensure that sustainability of LTRC in Ireland using best practices from Ireland and international systems. A case study of the largest COVID-19 intervention in the sector, the TAPs, will be undertaken to garner how such schemes could be used to inform future policies. 

The study design is described as having two main components. The first is a retrospective observational analysis of COVID-19 in LTRC homes in Ireland; the second, a review of funding, regulation, and governance structures in LTRC in Ireland and internationally. 

 With respect to the methods, the study is using routinely collected administrative datasets from HIQA, the NTPF and the HSE to examine characteristics (including size, provider-type, and ownership structure) of LTRC homes in Ireland. Population data will be applied to compare relative supply for people aged 65 years and older across the country. Data from the HPSC’s Computerised Infectious Disease Reporting (CIDR) database and from the HIQA bed register is being used to examine the association between LTRC characteristics and COVID-19 outbreaks and deaths. The study will undertake a review of funding models, regulation and governance structures in Ireland and identify those in other jurisdictions that could offer best practice for Ireland. Finally, the study is examining the Temporary Assistance Payment Scheme (TAPS) using data on TAPS from the Department of Health and the analysis of the operation of the scheme, including costs, and association with mitigating COVID-19 outbreaks and deaths.

 This is an important study, especially given the vulnerabilities of LTRC settings that have been exposed by the COVID-19 pandemic. It is an ambitious study, comprising four main components. Limited information is available on LTRC settings in Ireland and the overview of LTRC that will be provided by this study is much needed. The use of routinely collected administrative datasets in this study is to be welcomed. The study will make an important contribution to our understanding of the association between LTRC characteristics and COVID-19 outbreaks and deaths in Ireland. As the authors state, limited analysis has taken place on this subject in Ireland. This study will help to address this evidence gap and is important for that reason, as well as making an important contribution to the emerging international evidence on this subject. The examination of TAPS provides an interesting case study of one of the key ways in which the Irish government sought to limit the negative impacts on LTRC settings of the COVID-19 pandemic. The study has the potential to appraise current routine data collection on LTRC settings in Ireland and identify gaps that need to be addressed to strengthen future LTRC planning.

 Below, I have made a number of suggestions and comments for the authors to consider. I have also raised a number of questions prompted by my review of the study protocol. It may be beyond the scope of the study to address some of these questions, but I have included them here in any case for the authors’ consideration. 

**Specific suggestions/comments/questions:   **

- Page 3: It may be worth reporting estimates showing that most residents in LTRC are people with dementia, given the later points on issues with the current funding model failing to recognise and compensate for residents with complex needs (p. 4) and data limitations on residents (p. 5).
- Page 3: It would be useful to note that residents of private and voluntary nursing homes regularly incur additional charges not covered by the fees agreed between the nursing home and the NTPF.
- Page 3: The discussion on factors associated with COVID-19 outbreaks in Ireland relies solely on the analysis undertaken by HIQA and HPSC (2021). The Interim Report on COVID-19 in Nursing Homes prepared by the Oireachtas Special Committee on the COVID-19 Response (Houses of the Oireachtas, 2020) identified a range of factors that potentially contributed to COVID-19 outbreaks and deaths in LTRC settings and made a set of recommendations. ^1^ Yet, the Oireachtas Committee report is not mentioned which, it would seem to me, is a notable omission. The discussion would be enhanced by including reference to this report and key evidence gathered by this Committee. The Committee also identified important structural issues as having contributed to the spread of outbreaks in the LTRC sector in Ireland and highlighted several concerns. 
- Page 5: Sustainability is a core concept in this study, but an explicit definition is not given. I understand sustainability to be a multi-dimensional concept but appreciate that there is no standard definition. Is it an objective of the study to develop the concept of sustainability? I am further prompted to ask this question because, in the section on ‘Aims of this research’, it is stated that ‘sustainability will be considered from a number of perspectives including the resident and their family, the LTRC workforce, LTRC providers and the State.’
- Page 5: While the study design is described as having two main components, it would seem to me that it has four main components. The overview of LTRC in Ireland is an important and distinct component in its own right, as is the case study of TAPs.
- Page 5: Under Objective 1 - Overview of LTRC in Ireland, it is stated that ‘Comparative descriptive statistics will be used to describe differences in LTRC homes by size, provider-type, and other relevant characteristics identified within the data’. It would be very helpful to know what are the ‘other relevant characteristics’, especially given that this information is central to the second objective of the study, which is to examine the association between LTRC home characteristics and COVID-19 outbreaks and deaths.   
- Page 5: Is there an opportunity in this study to present an analysis of regional variation in LTRC supply with respect to people under 65 years of age? Will people under 65 years of age be included in the subsequent analysis examining COVID-19 in LTRC and TAPS? 
- Pages 5-6: In examining the association between LTRC home characteristics and COVID-19 outbreaks and deaths, the study will identify if particular types of homes were more or less likely to experience a COVID-19 outbreak and deaths. However, it appears that, due to data limitations, the analysis will only be able to provide information on the characteristics of LTRC homes according to provider type, size and location. Is this the case? This is not altogether clear. Earlier, it is stated on page 4 that this study is seeking to build on the international literature. Since there are the wide-ranging factors and conflicting evidence, will other factors that have been found to be associated with COVID-19 outbreaks and deaths in LTRC settings be included, if possible, in the analysis such as staffing (employment type, staffing levels), infection and prevention control compliance and physical premises. Furthermore, will it be possible in this study to examine factors such as those identified by the Oireachtas Special Committee on the COVID-19 Response including the discharge of patients from hospital to nursing homes; PPE levels and oxygen/fluids deficit; level of single occupancy rooms; and clinical oversight within LTRC settings? If not, could the authors comment further on data limitations covered on page 6. Please note that I am not an expert in the statistical data analysis that will be used to examine the association between LTRC characteristics and COVID-19 outcomes. 
- Page 6: Objective 3 – Sustainability of LTRC, appears to combine two distinct objectives, one on identifying challenges to the sustainability of the LTRC sector and a second on a case study of TAP. I believe there would be merit in splitting these into two separate objectives. 
- Page 6: Could the authors provide some clarity about the knowledge users and stakeholders with whom the researchers will work? In the section ‘Aims of this research’, it is stated that ‘In this project sustainability will be considered from a number of perspectives including the resident and their family, the LTRC workforce, LTRC providers and the State’. Are these the knowledge users and stakeholders? How do the researchers plan to engage and work with knowledge users?
- Page 6: A focus of the study is on what funding models, regulations, and governance structures can be identified as best practice in other jurisdictions that could be used to ensure high quality and safe care for LTRC residents in Ireland. What are the other jurisdictions that will be included in the review and how will these jurisdictions be selected?
- Page 6: With its focus on funding models, will the study be identifying financial packages opted for during the COVID-19 pandemic in other jurisdictions and the costs incurred, and comparing these with TAPS? In Germany, for example, LTRC settings incurring additional costs or loss of revenue due to the COVID-19 pandemic were reimbursed. However, steps were also taken by the German government to improve care workers’ wages (Lorenz-Dant, 2020), ^2^ an intervention that was not adopted in Ireland. Are there particular lessons to be learned from jurisdictions such as Finland, Norway, Germany and Denmark that had the lowest COVID-19 death rates among LTRC residents (Comas-Herrera *et al*., 2021)?
- Page 6: The study is examining whether TAPS was associated with lower rates of COVID-19 outbreaks and deaths. While TAPS was the largest intervention in financial terms, there were multiple components or elements in the state’s response such as the Area Crisis Management Teams, COVID-19 response teams and test and tracing in nursing homes. Will these other components be taken into account in the analysis of the correlation between TAPS and rates of COVID-19 outbreaks and deaths? Again, please note that I am not an expert in the statistical data analysis that will be used to examine the extent to which TAPS was associated with lower rates of COVID-19 outbreaks and deaths.  
- Language: Use of the term ‘patients’ versus ‘residents’ – There are three places (on pp. 3 and 6) in the article where the term ‘patients’ is used, and, in my view, the term ‘residents’ would be preferable.

Is the study design appropriate for the research question?

Yes

Is the rationale for, and objectives of, the study clearly described?

Yes

Are sufficient details of the methods provided to allow replication by others?

Yes

Are the datasets clearly presented in a useable and accessible format?

Not applicable

Reviewer Expertise:

Ageing, dementia and disability; long-term care; social policy analysis. I am not an expert in the statistical data analyses that will be used in this study to examine the association between LTRC characteristics and Covis-19 outcomes and whether TAPS was associated with lower rates of COVID-19 outbreaks and deaths.

I confirm that I have read this submission and believe that I have an appropriate level of expertise to confirm that it is of an acceptable scientific standard.

## References

[1] Houses of the Oireachtas Special Committee on Covid-19 Response (2020) Interim Report on Covid-19 in Nursing Homes. *House of the Oireachtas.* .2020; Reference source

[2] : Germany and the COVID-19 long-term care situation. *LTCcovid, International Long Term Care Policy Network, CPEC-LSE* .2020; Reference source

# Peer review: Reviewer response for version 1

**DOI:** 10.21956/hrbopenres.14783.r31867

**Published:** 2022-05-04

**Authors:** Brendan Kennelly

**Affiliations:** 1 Health Economic and Policy Analysis Centre, National University of Ireland Galway, Galway, Ireland

The project outlined in this protocol has three key objectives: (i) to describe and analyse the characteristics of Long-Term Residential Care (LTRC) homes across Ireland; (ii) to examine the association between LTRC home characteristics and COVID-19 outbreaks and deaths and (iii) to identify challenges to the sustainability of the LTRC sector within a COVID-19 environment and beyond.

 The research seeks to identify what funding models, regulations, governance structure, etc can be used to ensure high quality and safe care for LTRC residents.

 This is an important research issue. The protocol is well written and I am confident that the research will contribute significantly to better policy development and analysis.

- P. 3: “94% of LTRC residents are aged 65 or older”. It would be useful to know what percentage are 75 or older. The population in an area aged 75 or older may be a more useful proxy for demand for LTRC than the population aged 65 or older.
- P. 3: “From March 2020 to end of November 2021, 62% of COVID-19 deaths were linked to outbreaks in nursing homes.” It would be useful to give a reference for this. Also, could you clarify what ‘linked’ means? Does it for example include people who acquired COVID-19 in a nursing home but dies elsewhere? Does it include people who acquired the virus in hospitals or the community but died in a nursing home?
- That so many COVID-19 deaths were linked to outbreaks in nursing homes is a very striking statistic. I think it would be useful to break it down into, for example, 3-month periods or into ‘waves’ to see if the percentage of deaths linked to LTRC changed from the beginning of the pandemic to the period after vaccines became available. On page 6, you state that you may do econometric analysis by wave which I think is a very good idea. It is also worth remembering that the quality of data on cases was poor in the first couple of months of the pandemic so it would be useful to do some sensitivity analysis by excluding that period.
- P. 5: Reference is made to obtaining “Data on COVID-19 outbreaks and deaths in LTRC care between 28th February 2020 and 31st March 2021”. I acknowledge the benefits of using the same data as in the HIQA/HPSC analysis but would it not also be useful to use all the available data?
- P. 6: The analysis of the TAPS data will be very interesting. It is difficult to comment on this section because I have no idea what proportion of the nursing homes applied for or received TAPS funding. I am sure that the authors will be cautious about interpreting any associations between TAPS funding and outbreaks or deaths. It might be interesting to explore if the presence and nature of TAPS funding could be used as additional explanatory variables in the analysis in Section 2.

Overall, I think that the analysis of the TAPS scheme is sufficiently interesting and important that it merits its own section and objective. If the third objective were to focus on the TAPS scheme, then the fourth section could be more clearly focused on the sustainability of the sector in light of its characteristics and outcomes during the pandemic.

 Without knowing the research results or the implications drawn from the results, it is a little difficult to comment further. I would be a little cautious about how much weight to place on a specific case study in analysing funding models, regulations and governance structure. Is there, for example, a plausible counter factual scenario for the LTRC sector in Ireland pre-pandemic that would have resulted in much different outcomes as regards mortality, morbidity and quality of life more broadly?

 One possible approach that might be useful would be to analyse three issues separately whilst recognising their interdependence. One issue is how can the LTRC be better prepared for a future pandemic. A second issue is how the LTRC sector should be structured so as to improve the current lives of residents and staff. And the final issue is how the LTRC sector should be structured in the medium- and long-term given the likely increase in demand for LTRC due to demographic and other factors.

Is the study design appropriate for the research question?

Yes

Is the rationale for, and objectives of, the study clearly described?

Yes

Are sufficient details of the methods provided to allow replication by others?

Yes

Are the datasets clearly presented in a useable and accessible format?

Yes

Reviewer Expertise:

Health economics

I confirm that I have read this submission and believe that I have an appropriate level of expertise to confirm that it is of an acceptable scientific standard.
